# Enhancing Secure Messaging in Electronic Health Records: Evaluating the Impact of Emoji Chat Reactions on the Volume of Interruptive Notifications

**DOI:** 10.1055/s-0044-1788621

**Published:** 2024-07-25

**Authors:** John Will, William Small, Eduardo Iturrate, Paul Testa, Jonah Feldman

**Affiliations:** 1 MCIT Department of Health Informatics, NYU Langone Health, New York, New York, United States; 2 Department of Medicine, NYU Grossman School of Medicine, New York, New York, United States; 3 Ronald O. Perelman Department of Emergency Medicine, NYU Grossman School of Medicine, New York, New York, United States; 4 Department of Medicine, NYU Grossman Long Island, School of Medicine, Mineola, New York, United States

**Keywords:** secure messaging, EHR, communication, clinician burden, alert fatigue

## Abstract

**Background** Electronic health record secure messaging (EHRSM) is an increasingly utilized tool for communication among clinicians. However, there is concern about the growing quantity of disruptions it presents via interruptive notification.

**Objectives** The primary aim of this study is to assess whether introducing emoji reactions, which do not trigger push notifications in EHRSM, can alleviate the burden of interruptive notifications. The second aim is to use messaging notification metadata to identify subgroups that might benefit from targeted interventions to aid the adoption of this innovation.

**Methods** We implemented the emoji reaction feature into EHRSM across a large academic health system. We evaluated the volume of push notifications 11 weeks before (pre-emoji period) and after (post-emoji period) introducing emoji reactions in EHRSM. Notification metadata was categorized by user type, and users were stratified based on notification volume.

**Results** There were 1,387,506 fewer push notifications in the post-emoji period (a decrease of 4.7%). Subgroups of users with increasing mean daily push notifications in the pre-emoji period were associated with decreasing mean daily push notifications in the post-emoji period. Among the eight user subgroups, six experienced a significant reduction in interruptive notifications, with the pharmacy and “other” subgroups not observing a reduction. Users in the top quartile of notification volume saw the greatest reduction in burden across each user subgroup.

**Conclusion** Integrating emoji reactions into EHRSM across a large academic health system significantly reduced the burden of push notifications among EHRSM users. Utilizing messaging notification metadata allowed us to identify subgroups that require additional intervention.

## Background and Significance

Electronic health records (EHR) secure messaging (EHRSM) is an increasingly utilized tool for clinician–clinician communication. 1 Providers prefer communicating via texting-capable messaging tools rather than alphanumeric pagers due to perceived improvements in efficiency, 2 but find they may increase interruptions via frequent push notifications. 3 4 A push notification is a popup message that can appear on the lock screen of a mobile device, on an unlocked device screen regardless of the application in use, or on a desktop within the EHR application. In an analysis of patient safety events, sensitive patient tasks such as order management and medication administration were commonly interrupted events resulting in errors. 5 Another analysis of interruptions in an intensive care unit found human interruptions, such as side conversations and questions, accounted for 71.1% of observed interruptions. 6 In the same analysis, technological interruptions, specifically, computer alerts, pagers, and cell phones, were associated with patient safety events. 6

Notifications from EHRSM can pose a risk to patient safety beyond the interruption of the activities themselves. Excessive notifications are a risk to patient safety due to the impacts on the clinician's cognitive load. 7 Past research has shown that increased cognitive load is positively associated with medical errors. 8 Designers should consider how EHRSM contributes to clinician cognitive load and how that cognitive load impacts patient safety.

EHRSM and associated notifications can also negatively impact clinician well-being. A qualitative analysis of nurses and residents found increased stress resulting from increased communications via EHRSM. 9 Growing volumes of patient–clinician communications via EHRSM have been associated with burnout. 10 Despite recognizing the risks to patient safety, cognitive burden, and clinician burnout, no studies we found investigated interventions to reduce notifications from EHRSM.

Although no intervention studies exist, recent publications have highlighted the potential of using EHRSM metadata to begin to understand patterns of clinical communication behaviors and their impact. 1 3 11 At our institution, we have described using metadata to examine EHRSM communication by volume, by hour of the day, and by provider role of both message senders and recipients. 11 Although institutional policies limit how long certain data are stored, 11 we have developed strategies to collect relevant and necessary information. This analysis of metadata provides a framework for developing important process and outcome measures to identify user-level messaging burden and its impact on health care professional well-being and patient safety.

**Fig. 1:**
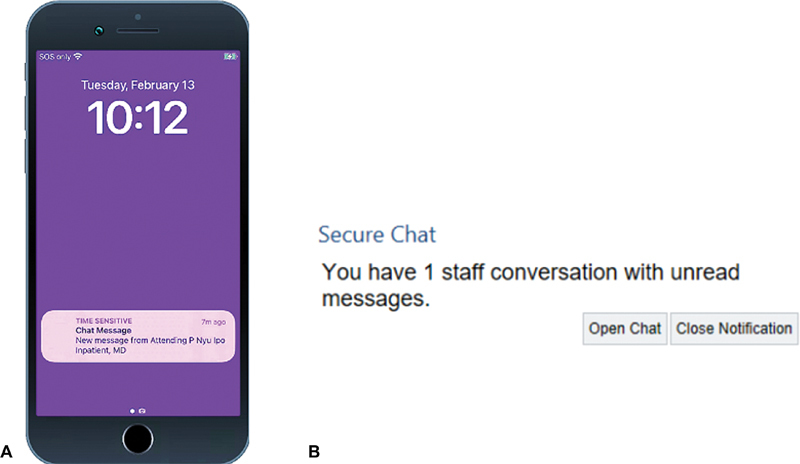
( **A** ) Example push notification display on a user's mobile device (2024 Epic Systems Corporation). ( **B** ) Example push notification display on desktop application (2024 Epic Systems Corporation).

Although emojis have been available in other communication platforms for years, Epic Systems (Verona, Wisconsin, United States) introduced emoji reactions into its EHRSM for use in clinician-to-clinician communications in November 2022. In addition to their diverse appeal and potential for communication enhancement, 12 emoji reactions allow for closing the communication loop without generating an interruptive push notification. Closed loop communication is important so the message sender can confirm the message reached the intended recipient. EHRSM push notifications are displayed on users' mobile devices ( Fig. 1A ) or desktop computers ( Fig. 1B ) unless they are actively in the EHRSM activity on either device. Mobile push notifications will either display on the lock screen or appear at the top, middle, or bottom of the user's screen depending on their notification settings. It will also trigger an audible alert if the phone is not silenced, in which case it will vibrate. The desktop notification defaults to displaying at the bottom right of the user's screen unless the user has moved the notification window.

**Fig. 2:**
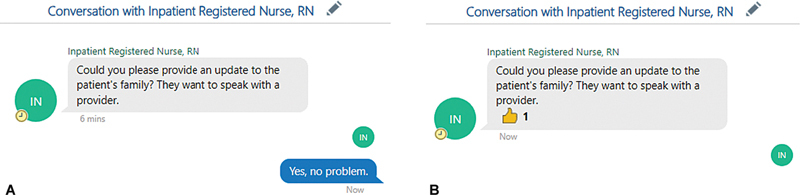
( **A** ) Electronic health record secure message prior to emoji reaction implementation with text response (2024 Epic Systems Corporation). ( **B** ) Electronic health record secure message after emoji reaction implementation with emoji response (2024 Epic Systems Corporation).

Prior to emoji reactions a text response was required if the recipient wanted to convey acknowledgment ( Fig. 2A ). After the introduction of emoji reactions, users could add an emoji response to an incoming message, acknowledging its receipt without generating an interruptive notification ( Fig. 2B ).

The primary objective of this study is to investigate whether the introduction of emoji reactions led to a reduction in interruptive EHRSM push notifications across our health system. The secondary objective is to use messaging notification metadata to identify subgroups that might benefit from targeted interventions to aid the adoption of this innovation. Our hypothesis is that our users, particularly those with the highest messaging burden like clinicians, would receive fewer push notifications in the period after introducing emoji responses to secure messages.

## Methods

This is a retrospective study conducted on EHRSM at NYU Langone Health (NYULH), a large, urban, academic health system comprising five hospitals, over 5,000 health care providers, and more than 500 ambulatory locations. We followed the STROBE (Strengthening the Reporting of Observational Studies in Epidemiology) combined checklist for reporting observational studies. 13

EHRSM was first introduced to NYULH users in 2017. Emoji reactions were made available to all health system users for all messages on May 21, 2023. The initial set of emoji reactions included a “thumbs up,” “heart,” a “laughing face,” a “sad face,” and a “surprised face.” The new EHR functionality was advertised in advance to all users via video preview in an electronic learning module on the health systems' learning management system, email communications, enterprise role-specific meetings, and at faculty and business meetings for 15 different service lines.

We extracted push notification data, including notification instants, recipients, and devices from Epic System's Clarity database using SQL Developer, 14 and exported it to analyze records from EHRSM created between March 5, 2023 and August 5, 2023. We classified the period from March 5, 2023 to May 20, 2023 as the pre-emoji reaction period and May 21, 2023 to August 5, 2023 as the post-emoji reaction period. For each 11-week period, we calculated the total and daily EHRSM push notification volume per user and examined the overall linear trend. For comparison to the year before, we also extracted push notification records from EHRSM between March 5, 2022 and May 5, 2022. Because of the ability for groups to send messages to one another, one message may result in multiple push notifications. Any user on the push notification record recipient list in the push notification record was considered to have received a push notification, regardless of receipt on desktop, mobile, or both types of devices. For our analysis, although multiple recipients are listed in a single record, they were qualified as having received their own distinct push notification on the resulting device(s). Thus, one push notification record with five unique recipients would result in five push notifications for analysis. We also measured inpatient hospital admission volume (as defined by number of unique admissions) and completed appointment volume to compare NYULH activity in the pre- and post-emoji periods. Hospital admission and appointment volume data were extracted from Epic SlicerDicer (Caboodle database) and organized by date into the pre- or post-emoji period.

Push notification data were measured at the user level, and users were grouped by user type. User type was determined by the “provider type” setting in the user's linked provider record in the EHR. The “provider type” is an indicator of the role they serve in caring for the patient (e.g., physician, resident, nurse, respiratory therapist, etc.) and are derived from prior research, 1 11 which aligns with operational practice at our institution. All NYULH EHR users can send and receive messages on their desktop computers, and if the Epic mobile application (i.e., Haiku, Canto, or Rover) is installed, their mobile devices. Users who did not receive at least one push notification during the pre- and post-emoji reaction periods were excluded from the analysis.

We used paired-sample *t-* tests to identify significant differences in notifications in the pre- and post-emoji reaction periods. We performed linear regression analysis to test for a relationship between push notifications in the pre-emoji period and changes in the post-emoji period. Because use of EHRSM and subsequent push notification volume may vary by individual, we further grouped individuals within each user type subgroup into the top 25% of push notification recipients (PNR) and bottom 75% of PNR, based on the volume of push notifications received in the pre-emoji period ( Fig. 1 ). All analyses were performed in IBM SPSS Statistics Version 28.0.1.1. 15

## Results

**Fig. 3:**
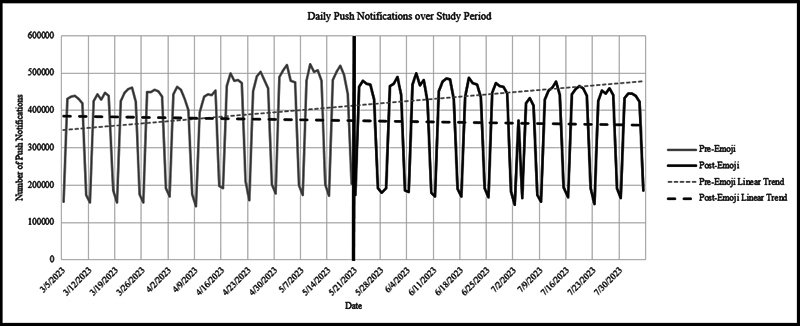
Daily push notification volume changes during the pre- and post-emoji periods.

There were 1,387,506 fewer push notifications resulting from EHRSM in the post-emoji reaction period ( *n* = 27,938,526) than in the pre-emoji reaction period ( *n* = 29,326,032), representing a 4.7% reduction in overall notifications and an average of 18,019 fewer notifications per day across our institution. Push notifications in the pre-emoji period were noted to be increasing by a mean of 855.2 notifications per day. This contrasts with a mean decrease of 163.3 notifications per day in the post-emoji period ( Fig. 3 ).

Across all users ( *N* = 28,445), daily mean push notifications per user decreased by 0.6 notifications (from 13.4 to 12.8; *p* < 0.001). Residents, fellows, and interns saw the greatest reduction in daily mean push notifications during the post-emoji reaction period (from 21.6 to 17.6; *p* < 0.001) followed by case management and social work users (from 21.8 to 20.1; *p* < 0.001). Pharmacists, who were the users with the lowest overall notification volume, were the only group that saw an absolute, but nonsignificant, increase in daily mean push notifications in the post-emoji reaction period ( Table 1 ).

**Table 1:** Changes in volume of push notifications from electronic health records-based secure messaging ( *N* = 28,445)

User type	*n* (subgroup)	% of study population	Push notifications (pre)	Push notifications (post)	Difference (all)	% Mobile (pre)	% Mobile (post)	Mean daily per user (pre)	Mean daily per user (post)	*p* -Value a
Attending	3,938	13.8	3,011,446	2,897,252	−114,194	91.1	92.0	9.9	9.6	<0.001
Resident, fellow, or intern	1,548	5.4	2,573,767	2,097,907	−475,860	95.7	96.8	21.6	17.6	<0.001
Physician assistant or nurse practitioner	2,135	7.5	3,171,936	3,043,029	−128,907	88.2	88.3	19.3	18.5	0.002
Nurse	7,368	25.9	8,289,223	7,914,795	−374,428	76.4	78.5	14.6	14.0	<0.001
Case management and social work	734	2.6	1,231,894	1,135,447	−96,447	87.1	87.7	21.8	20.1	<0.001
Therapies	1,055	3.7	820,257	721,787	−98,470	87.4	85.7	10.1	8.9	<0.001
Pharmacy	412	1.4	725,578	765,160	39,582	42.1	40.5	22.9	24.1	0.076
Other b	11,255	39.6	9,501,931	9,363,149	−138,782	55.7	57.1	11.0	10.8	0.059

a *p* -Value for paired samples *t* -test comparing the mean daily push notifications per user in the pre- and post-emoji periods.

b Other users may include registered dietitians, phlebotomists, technicians, etc. It may also include individuals without a specified role.

Using linear regression, we used the mean daily push notifications in the pre-emoji period to predict the difference in mean daily push notifications that occurred in the post-emoji period. Regression results showed a significant, negative relationship (β = −0.092, *p* = 0.001). A total of 6.8% of the variance was explained by the mean daily push notifications in the pre-emoji period [R ^2^ = 0.068, F(1, 28443) = 2076.0, *p* = 0.000]. After adjusting the regression to account for the user type, variance remained similar [R ^2^ = 0.069, F(2, 28442) = 1050.0, *p* = 0.000] and the significant, negative relationship persisted (β = −0.091, *p* = 0.000).

**Fig. 4:**
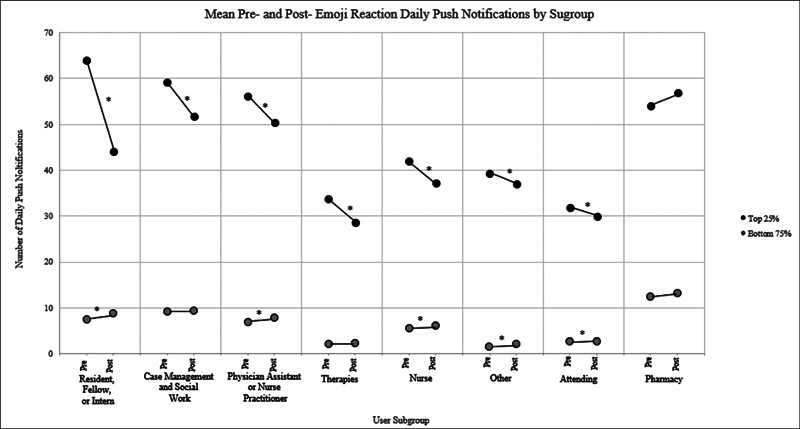
Changes in mean daily push notifications by user-type subgroup and push notification recipient subgroup ( *N* = 28,445). * *p* < 0.05.

Changes in mean daily push notifications varied by user type subgroup and again by the volume of push notifications received in the pre-emoji period. In all user-type subgroups with the exception of pharmacy, the top 25% of PNR in the pre-emoji period saw decreases in push notifications in the post-emoji period. The top 25% of PNR in the residents, fellows, and interns subgroup had the greatest reduction in mean daily push notifications (a decrease of 19.8 push notifications or 31.0%). The top 25% of PNR in the case management and social worker; physician assistants and nurse practitioner; therapies; and nurse subgroups all saw decreases in push notifications (12.5, 10.3, 15.4, and 11.2%, respectively). The bottom 75% of PNR in all subgroups had increases in mean daily push notifications, although not all were significant ( Fig. 4 ).

In the year prior to the study, a 0.1% increase in push notifications was seen over the time periods of March 5, 2022 to May 20, 2022 to May 21, 2022 to August 5, 2022. In contrast, there was a decrease in push notifications of 4.7% between the study time periods of March 4, 2023–May 20, 2023 to May 21, 2023–August 5, 2023 ( Table 2 ). Hospital admissions across NYULH were 0.7% higher in the post-emoji period with 189 more admissions. The volume of ambulatory completed visits decreased by 5.6%, with 123,902 fewer completed visits in the post-emoji reaction period.

**Table 2:** Push notification volume and percent change between the pre- and post-emoji periods for 2022 and 2023

Year	Dates March 5–May 20	Dates May 21–August 5	% Change
2022 (baseline)	19,705,612	19,720,661	0.1
2023 (study period)	29,326,032	27,938,526	−4.7

## Discussion

As health care organizations transitioned from pagers to mobile phones, communication interruptions increased, 16 raising concerns about patient safety and clinician well-being. The results of our analysis show that the adoption of EHRSM emoji reactions could be a promising communication tool that enhances conversations 12 by conveying understanding and acknowledgment while mitigating interruptions that could negatively impact patient care. 17 Significant reductions in push notifications were seen in our health system in the post-intervention period, contrasting with a slight overall increase in push notifications between the same dates in the year before the study period. Further, our organization had a trend of increasing push notifications in the pre-emoji period, potentially due to the continued adoption of EHRSM among different users and for new communication workflows. This trend was reversed after the introduction of the emoji reaction.

By leveraging the metadata on push notifications, including creation instants, recipients, recipient devices, and notification types at our institution, we identified that pharmacy users and the bottom 75% of PNR across all subgroups may benefit from additional interventions to promote the use of newly available technology, or more qualitative analysis to understand barriers to adoption. The lack of reduction among pharmacy users may be because pharmacy is segmented from other health system users and may be later to adopt new features when communicating within their department. Individuals in different departments communicating with pharmacy may also feel that emoji reactions do not properly convey message meaning for these clinical scenarios. In general, the bottom 75% of PNR may be less exposed to newly available technology features because of limited use, and our organization is currently pursuing targeted educational interventions (tip sheet distribution, roadshows, inline decision support) to promote emoji adoption and reduce interruptive notifications.

Unequal use of emoji reactions throughout NYULH may contribute to disproportionate changes in notification volume. This could be due to perceptions of appropriateness or professionalism, cultural differences among message senders/receivers, age, variance in clinical workflows, or indecisiveness about the meaning conveyed when applying a certain emoji. To encourage additional, appropriate use of emoji reactions on EHRSM, health systems could provide guidelines with communication best practices that promote emoji reactions in appropriate scenarios to convey message acknowledgment without adding to the notification burden. Appropriateness of use would need to be determined by the organization, and discussion is underway at our organization about how to explicitly incorporate emoji use etiquette in our existing clinical communications guidelines.

More qualitative research is required to understand if clinicians perceive the decrease in push notifications as a reduction in burden, or if there is a minimum percentage decrease for the reduction in notifications to be noticeable. Additionally, it is unclear why the push notification contributes to a clinician's cognitive load. Additional research should examine if it is the content of the notification, the toggling between activities, or some other unidentified effect related to the push notification that adds to the clinician's cognitive load. Future studies could also assess if there are additional measures beyond emoji reactions needed to continue reducing EHRSM push notifications. Behavior modifications, such as encouraging message length or frequency standards, could be explored to limit the number of EHRSM being sent to avoid unnecessary push notifications. From a technical design perspective, EHRs could adjust push notifications to allow for “batching” of nonurgent EHRSM occurring within seconds of each other. EHRs could also allow for settings that hold notifications during tasks that require focus such as nursing medication administration or provider order entry while allowing for notifications during other activities like note or report viewing, based on which EHR activity is being accessed. Artificial intelligence-based technology could also be utilized to bring EHRSM notifications to light at the right moment in a clinician's workflow.

## Limitations

This is a single-center study and is limited in its generalizability to other institutions that may be considering implementing emoji reactions in EHRSM. By nature of the study design, the pre- and postperiods are not identical in terms of factors that may affect EHRSM use. We were unable to account for patient workload and acuity during the study period, which could impact the use of EHRSM by individuals, although patient admission slightly increased in the postperiod and completed appointments slightly decreased. It is possible users were more actively engaged in EHRSM during any period of the study, which we were unable to measure but would have impacted the generation of push notifications. While all users were given similar notice that the emoji reaction feature was available, we were unable to verify who had knowledge of the feature availability. Although we only included users who had a minimum of one push notification in both the pre- and post-emoji periods, our study does not account for users who may have transitioned roles or changed work schedules, impacting EHRSM use. Further, we were unable to collect demographic characteristics beyond their clinical role that may impact emoji reaction use. Lastly, it is possible there are initiatives within the hospital that either promote or discourage the use of EHRSM, and emoji reactions, that we are not aware of and thus did not account for in the data.

## Conclusion

In conclusion, the integration of emoji reactions resulted in a statistically significant decrease in the volume of EHRSM push notifications within our health care system. The feature particularly benefited users who initially received the highest number of notifications. Messaging notification metadata proved valuable for pinpointing subgroups that may benefit from further intervention, by examining who is still sending text replies in EHRSM and the rationale. As a next step, exploring the potential hindrances to adopting this feature and other determinants of EHRSM notification volume is needed. Further research should examine the possible impact of push notifications on clinicians' cognitive load and, consequently, patient safety.

## Clinical Relevance Statement

Communication among clinicians benefits from acknowledgment by message recipients to close the loop on message threads. Emoji reactions are already ubiquitous in informal settings. This feature can be cross applied to the clinical setting to affirm receipt and understanding of a communication without further impacting the cognitive burden placed on clinicians.
